# Metastatic tropism of molecularly defined clear-cell renal cell carcinoma clusters

**DOI:** 10.1172/JCI195288

**Published:** 2026-05-15

**Authors:** Gaelle Haddad, Junyu Guo, Yin Xi, Emin Albayrak, Mahrukh Huseni, Habib Hamidi, Romain Banchereau, Edward Kadel, Sarita Dubey, Corey Carter, Payal Kapur, James Brugarolas, Ivan Pedrosa

**Affiliations:** 1 Department of Radiology, University of Texas Southwestern Medical Center, Dallas, Texas, USA.; 2 Genentech, San Francisco, California, USA.; 3 Department of Pathology,; 4 Department of Urology,; 5 Kidney Cancer Program,; 6 Department of Medicine, and; 7 Advanced Imaging Research Center, University of Texas Southwestern Medical Center, Dallas, Texas, USA.

**Keywords:** Clinical Research, Oncology, Public Health, Cancer, Diagnostic imaging, Molecular genetics

## Abstract

**BACKGROUND:**

The relationship between molecular subgroups in clear-cell renal cell carcinoma (ccRCC) and metastatic tropism is poorly understood.

**METHODS:**

We analyzed over 5,000 metastatic sites from 305 treatment-naive ccRCC patients in the IMmotion150 phase II clinical trial, where patients were randomized to atezolizumab, atezolizumab/bevacizumab, or sunitinib.

**RESULTS:**

Angiogenic tumors (clusters 1 and 2) had a higher rate of pancreatic (21% vs. 6.9%; *P* = 0.002) and lower absolute number of lymph node (2.5 vs. 4.2; *P* = 0.006) metastases. In contrast, proliferative tumors (clusters 4 and 5) exhibited a higher absolute number of lymph node metastases (5.5 vs. 3.5; *P* = 0.019). Patients with pancreatic metastases receiving sunitinib had higher odds of overall response (OR, 7.13; 95% CI, 1.81–28.07; *P* = 0.0049) and longer progression-free survival than those without pancreatic metastases (*P* = 0.02).

**CONCLUSION:**

ccRCC metastatic tropism relates to molecular clusters that predict response to therapy for tumors that metastasize to the pancreas.

**TRIAL REGISTRATION:**

ClinicalTrials.gov NCT01984242

**FUNDING:**

NIH grants R01CA154475 and P50CA196516.

## Abstract 2

Comprehensive analysis of the kidney cancer tumor burden confirmed that the anatomic distribution of metastasis relates to molecular clusters associated to response to specific therapies.

## Introduction

Renal cell carcinoma (RCC), the most common primary renal malignancy, causes approximately 175,000 deaths annually worldwide (1). Clear-cell RCC (ccRCC) is the most common histopathological subtype and the most common cause of metastatic RCC (2, 3); it is known to metastasize to virtually any organ (4). The treatment of metastatic ccRCC has evolved over the last 2 decades based on a deeper understanding of the molecular basis of the disease (5–7). First, antiangiogenic agents (AAs) targeting the HIF/VEGF pathway, such as tyrosine kinase inhibitors (TKIs), were introduced (5). More recently, immuno-oncology (IO) therapy exploiting programmed cell death protein 1/ligand 1 (PD-1/PD-L1) and cytotoxic T-lymphocyte–associated protein 4 antibodies have been developed and offer unprecedented oncologic outcomes (8). Indeed, current clinical guidelines for metastatic ccRCC favor the use of combination therapy that include IO drugs, although single AA therapy is still recommended in selected patients (e.g., contraindication to IO). Unfortunately, the administration of any therapy to patients who are unlikely to respond exposes them unnecessarily to side effects, including fatal complications in up to 1% of patients receiving IO (e.g., Gillen-Barré syndrome, myocarditis, agranulocytosis, etc.) and substantial deterioration of their quality of life in virtually all patients receiving AA (9, 10).

IMmotion150 (ClinicalTrials.gov NCT01984242) was a randomized phase II clinical trial that compared disease response of 305 patients with treatment-naive metastatic RCC (mRCC) to atezolizumab (PD-L1 antibody) versus atezolizumab plus bevacizumab (anti-VEGF antibody) versus sunitinib (TKI) (11). Tumors had clear-cell histology and/or sarcomatoid differentiation. This study was followed up by the IMmotion151 phase III clinical trial, which included 823 mRCC patients randomized to either atezolizumab/bevacizumab versus sunitinib (12). Transcriptomic analyses in IMmotion151 revealed 7 distinct molecular subsets, which were associated with response to AA and IO therapy and were validated in the previous IMmotion150 study (12, 13). For instance, patients in clusters 4 and 5 (T-effector/proliferative and proliferative) exhibited a higher response rate to atezolizumab/bevacizumab than to sunitinib. In contrast, patients in clusters 1 (angiogenic/stromal) and 2 (angiogenic) exhibited more robust responses to sunitinib compared with other clusters. Thus, molecular clusters may help optimize therapeutic regimens in mRCC. However, although promising, the implementation of transcriptome analysis is not without challenges.

More recently, the clinical significance of tumor tropism in mRCC has been proposed. mRCC patients with pancreatic metastasis have longer overall survival than those without pancreatic metastases (14–17). In a retrospective review of 31 mRCC patients with pancreatic metastases, we reported their indolent biology (with preferential mutation of *PBRM1* over *BAP1*), heightened angiogenesis, and an uninflamed stroma, likely underlying the good prognosis (18). Importantly, these patients exhibited sensitivity to AA but refractoriness to IO (18). Subsequent studies of a multi-institutional cohort involving more than 100 patients with pancreatic metastases found that PBRM1-deficient tumors were associated with preferential response to AA (19). However, the relation between molecular clusters and tumor tropism in mRCC is not well understood. Moreover, the clinical significance of the phenotypic presentation of mRCC in the entire body in the context of AA and IO therapies has not been reported. Here, we perform an extensive analysis of the IMmotion150 patients, including all visible metastases in each patient’s radiological studies prior to and after initiation of therapy, characterizing the anatomic distribution and tumor burden of over 5,000 metastases. We report a relationship between molecular clusters and tumor tropism and validate the presence of pancreatic metastases as an imaging biomarker of response to AA therapy.

## Results

### Baseline data.

Imaging studies for 305 patients were available for review (Supplemental Table 1; supplemental material available online with this article; https://doi.org/10.1172/JCI195288DS1). Two patients were excluded: 1 (sunitinib arm) due to withdrawal of consent before receiving therapy and 1 (atezolizumab arm) due to lack of measurable metastatic disease on imaging. Table 1 describes the baseline characteristics of the study cohort. The median age was 61 years (range 25–88) with 75.2% male patients. Overall, 243 (80.2%) patients had prior nephrectomies, only 3 of which were partial (1 from the atezolizumab arm and 2 from the sunitinib arm). Intent-to-treat progression-free survival (PFS) HRs for atezolizumab/bevacizumab or atezolizumab monotherapy versus sunitinib were 1.0 (95% CI, 0.69–1.45) and 1.19 (95% CI, 0.82–1.71), respectively (11).

### Anatomic distribution of metastatic disease.

A total of 5,164 individual metastatic sites were identified at baseline with an average of 17 metastases per patient (range 1–285). As the number of metastases in a specific organ increased, so did the overall tumor burden (cm^3^) in that organ, although the slope for lung metastases was higher than that for all other organs (i.e., greater number of metastases observed in the lung but smaller in size on average) (Supplemental Figure 1). Although lung metastases represented the highest count rate per organ for all molecular clusters, their relative contribution to the overall tumor burden was lower than that of lymph nodes, bone, and liver metastases.

**Figure 1:**
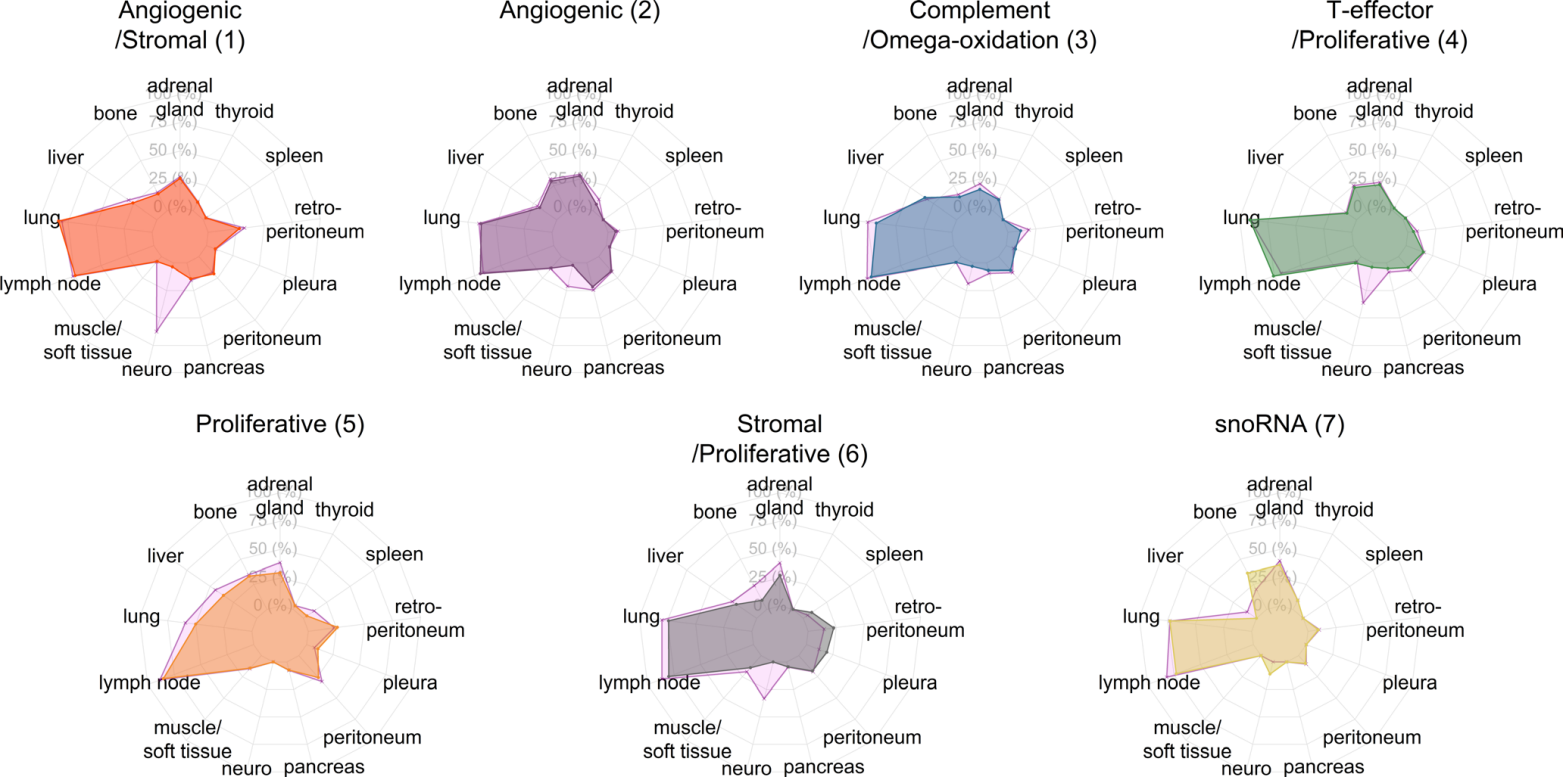
Prevalence of metastases by anatomic location at baseline and follow-up for each molecular cluster. Radar plots showing the frequency of metastases by anatomic site. Spokes correspond to a specific anatomic locations. Each graph displays the percentage of patients with metastasis in each anatomic location, where the inner circle represents no metastasis (0%) and the 4 concentric circles increasing in size represent 25%, 50%, 75%, and 100% of patients with metastases, respectively. The solid area with different colors indicates the distribution of metastases at baseline for each cluster. The light pink area indicates the distribution of metastases at follow-up imaging. At baseline imaging, the lungs and lymph nodes were the most common locations for metastases in all clusters. The lung was the most common site in clusters 1 and 4, whereas lymph nodes were the most common site in clusters 3 and 5. The frequency of metastases in lung and lymph nodes in clusters 2, 6, and 7 were almost identical, whereas lung metastases were less common in cluster 5. At follow-up imaging, the frequency of metastasis did not change for most anatomic sites, resulting in complete overlap between the colored and light pink areas, except for the increased metastases in the brain (clusters 1–4 and 6). Cluster 6 also exhibited increase metastases in bone, lung, and lymph nodes. Cluster 5 exhibited increased metastases predominantly in lung, liver, and adrenal glands. Neuro, CNS; snoRNA, small nucleolar RNA.

The percentage of patients with metastases, number of metastasis (tumor count), and total burden per organ (cm^3^) for each molecular cluster are shown in Figure 1 and Supplemental Figures 2 and 3, respectively. Brain metastases were more common at follow-up imaging and involved clusters 1, 4, and 6. However, out of 11 patients with new brain metastasis at follow-up MRI, 7 had no brain metastasis at a baseline CT (i.e., less sensitive test), 1 had brain metastasis at a baseline MRI, and 3 had no baseline imaging of the brain.

### Correlation with molecular clusters.

Next, we sought to compare metastasis patterns to tumor clusters. Tumor clusters were defined by RNA-seq (11). Overall, 42.2% of tissue samples were obtained from the primary tumor and 55.1% from a metastatic site (Supplemental Tables 2 and 3). The site of tissue sampling was unknown in 2.6% of patients. Importantly, biopsies accounted for 52.5% of the samples, and they likely reflect sites of disease that remain in the patient (unlike surgery specimens).

Over 50% of the patients from every cluster had lung and lymph node involvement at baseline and follow-up imaging. The percentage of patients with lung metastasis at baseline in the proliferative cluster 5 (50%) was statistically lower than that of the rest of clusters (74%; *P* = 0.008).

**Figure 2:**
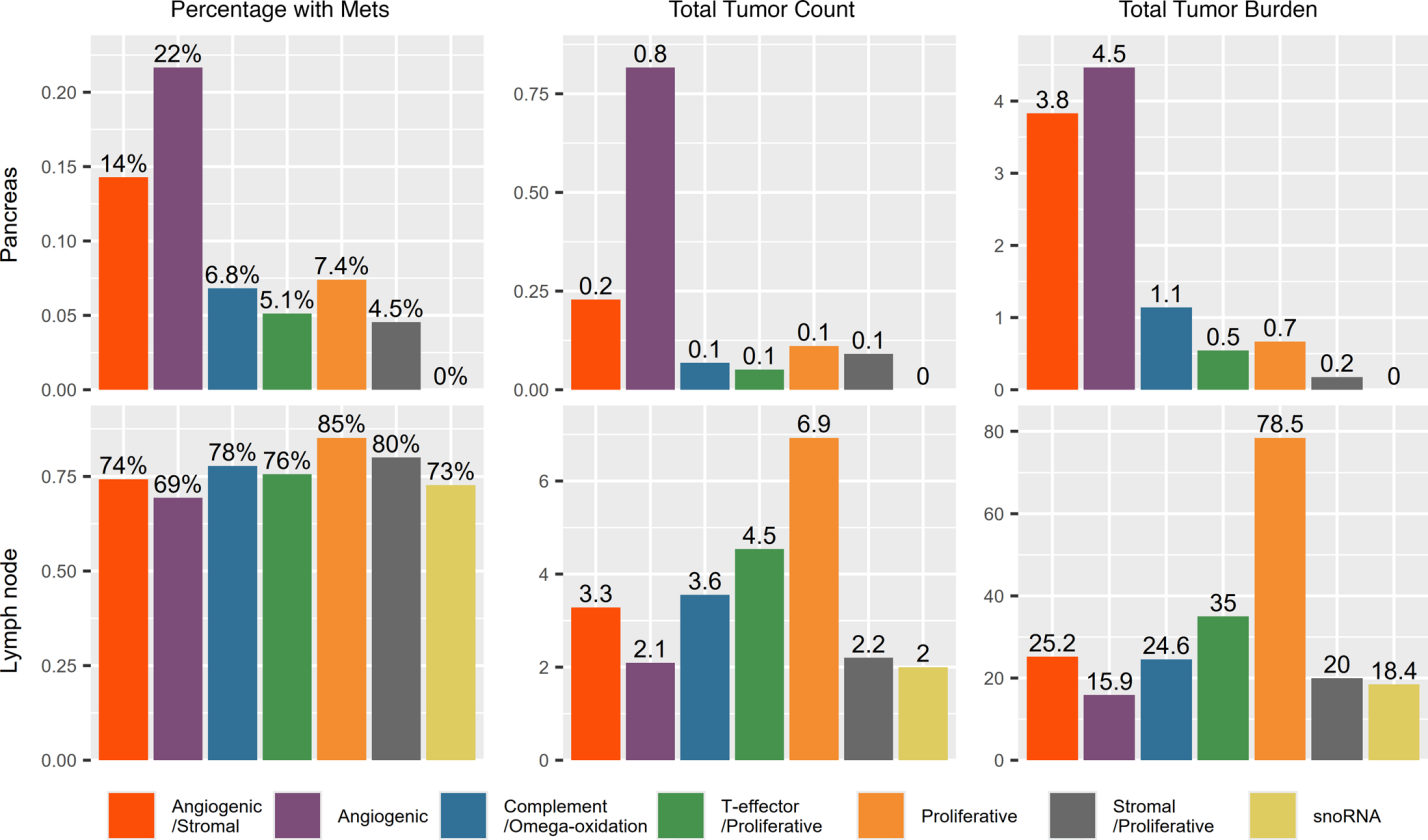
Pancreatic and lymph node metastases by molecular cluster. Bar charts illustrating the prevalence of metastases (Percentage with Mets), average of total tumor count, and metastatic tumor burden (cm^3^) of metastases at baseline in the pancreas and lymph nodes.

When compared with other clusters at baseline, angiogenic clusters (clusters 1 and 2) were associated with a significantly higher rate of pancreatic metastases (18.9% vs. 5.6%; *P* = 0.001), a greater tumor count in the pancreas (0.6 ± 1.9 vs. 0.07 ± 0.31; *P* < 0.001), and a higher tumor burden in the pancreas (4.2 ± 13.7 cm^3^ vs. 0.7 ± 3.4 cm^3^; *P* = 0.001) (Figure 2). Out of the 31 patients with pancreatic metastasis, 18 were in angiogenic clusters versus 8 in remaining clusters (clusters 3–7). The molecular cluster was not available in 5 patients. Angiogenic clusters also had a lower absolute number of lymph node metastases compared with the rest of the clusters (2.5 ± 3.7 lymph nodes vs. 4.2 ± 5.6 lymph nodes; *P* = 0.006). Proliferative clusters (clusters 4 and 5) exhibited a higher absolute number of lymph node metastases when compared with the rest (5.5 ± 7.2 vs. 2.7 ± 3.6; *P* = 0.012).

### Response to treatment.

We then evaluated the effect of the anatomic distribution of metastases on treatment response. Because tumor burden and tumor count were right-skewed, values were log_2_ transformed. A 1-unit increase therefore represents a doubling, and model coefficients were interpreted as the change in odds or hazard associated with each doubling of tumor burden or tumor count. The association between treatment response and pancreatic metastasis varied statistically for different treatment arms (*P* values for the interaction term = 0.017 [disease presence pancreas] and 0.011 [burden doubling pancreas]). The presence of pancreatic metastasis was associated with a significantly higher odds of complete or partial response with sunitinib (overall response [OR], 7.13; 95% CI, 1.81–28.07; *P* = 0.0049), but, if anything, atezolizumab trended in the opposite direction (OR, 0.11; 95% CI, 0.01–2.29; *P* = 0.166). Similarly, tumor burden in the pancreas was associated with response to sunitinib (OR, 1.78; 95% CI, 1.18–2.69; *P* = 0.006) but not to atezolizumab (OR, 0.58; 95% CI, 0.25–1.36; *P* = 0.21). Atezolizumab/bevacizumab was not associated with preferential response in patients with pancreatic metastases (OR, 0.75; 95% CI, 0.14–3.94; *P* = 0.74) or as assessed by tumor burden doubling (OR, 0.79; 95% CI, 0.48–1.30; *P* = 0.35) (Supplemental Figures 4 and 5).

**Figure 3:**
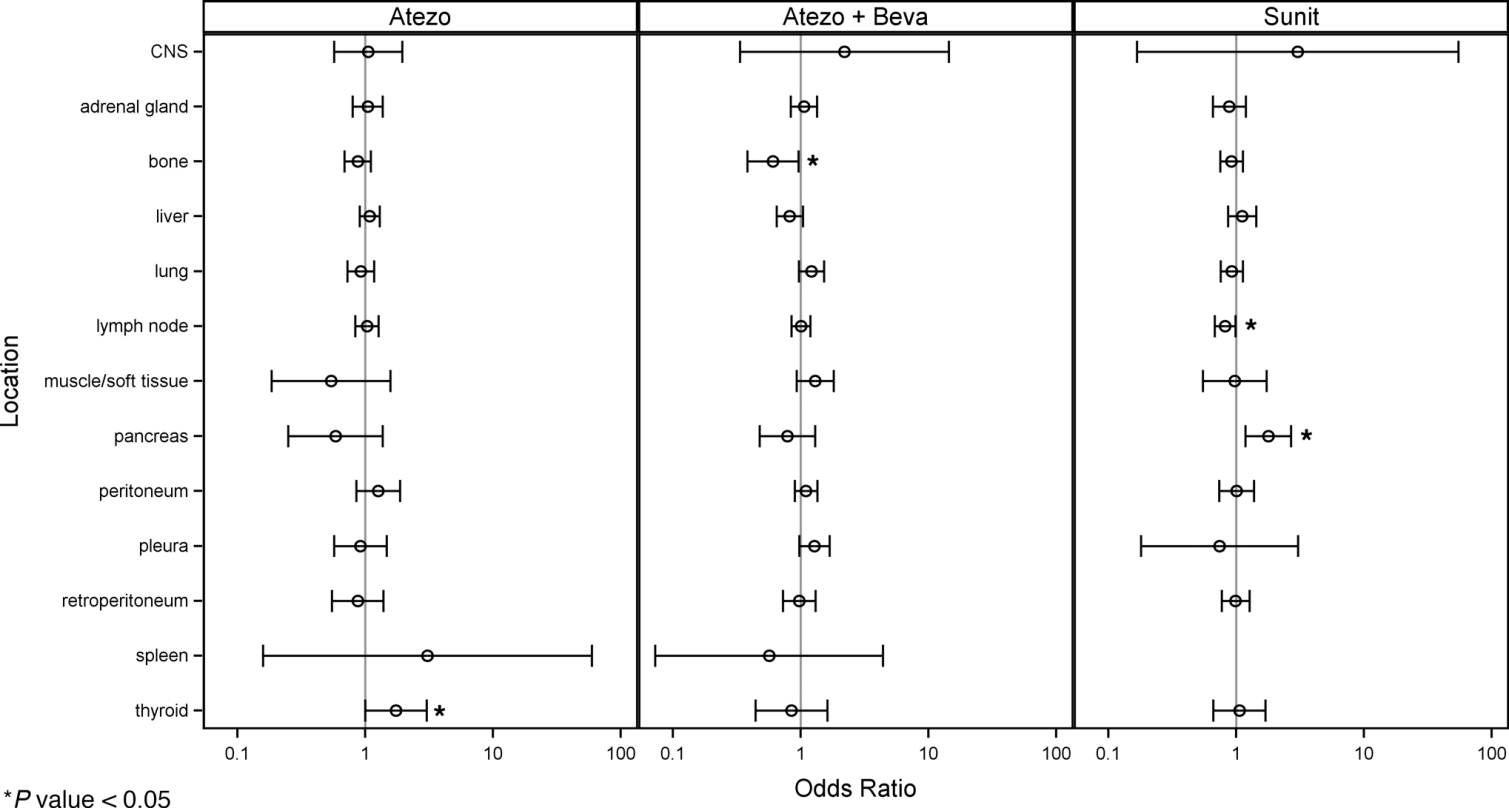
Response to treatment according to the total tumor burden in different organs. Estimated ORs (circles) and 95% CIs (brackets) for best response (CR/PR vs. SD/PD) based on total tumor burden in each anatomic location for each treatment arm. Higher odds represent higher likelihood of response. Atezo, atezolizumab arm; Atezo + Beva, atezolizumab plus bevacizumab arm; Sunit, sunitinib arm.

Patients with higher count or burden of bone metastases were associated with lower likelihood of complete or partial response across treatment arms. When averaged across treatment arms, the corresponding ORs were 0.44 (95% CI, 0.22–0.89; *P* = 0.023) for disease presence, 0.54 (95% CI, 0.34–0.87; *P* = 0.011) for a doubling of tumor count, and 0.82 (95% CI, 0.70–0.95; *P* = 0.007) for a doubling of tumor burden (Figure 3, Supplemental Figures 4 and 5, and Supplemental Table 4).

Patients with angiogenic cluster tumors had a statistically more favorable response to sunitinib than proliferative clusters (OR, 28.78; 95% CI, 4.53–182.99; Holm’s adjusted *P* = 0.003) and trended toward significance compared with the rest of the clusters (OR, 4.66; 95% CI, 1.53–14.19; Holm’s adjusted *P* = 0.053). However, a similar difference was not seen for patients with angiogenic cluster tumors 1 and 2 receiving atezolizumab or atezolizumab/bevacizumab (Table 2 and Supplemental Table 5).

### PFS.

**Figure 4:**
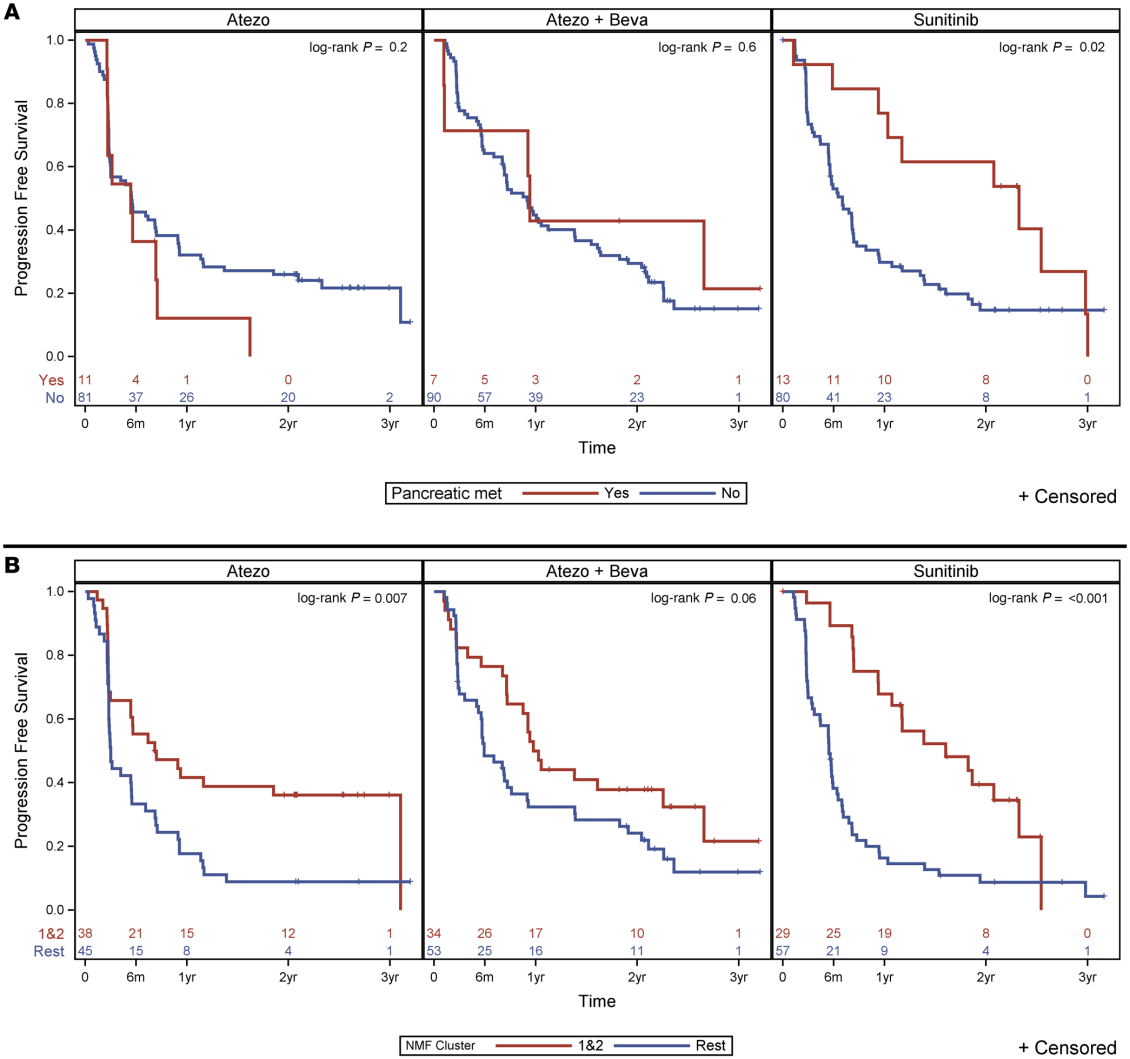
PFS in patients with and without pancreatic metastases and in clusters 1 and 2 versus the rest. Kaplan-Meier curves for patients in the 3 treatment arms with (red line) and without (blue line) pancreatic metastases at baseline (**A**) and for patients in clusters 1 and 2 (red) versus the rest (blue) (**B**). A statistically significant longer PFS was observed for patients with pancreatic metastases receiving sunitinib (log rank test, *P* value = 0.02). Atezo, atezolizumab arm; Atezo + Beva, atezolizumab plus bevacizumab arm.

The PFS of patients presenting with pancreatic metastases on sunitinib showed a trend toward being significantly higher than for those without pancreatic metastases (HR, 0.52; 95% CI, 0.27–1.01; *P* = 0.05). Additionally, the analysis of pancreatic doubling tumor burden indicated a similar trend (HR, 0.83; 95% CI, 0.69–0.998; *P* = 0.048) (Supplemental Table 6). A higher PFS in patients with pancreatic metastases treated with sunitinib was also visualized by Kaplan-Meier analysis (Figure 4). The median PFS for patients with pancreatic metastases on sunitinib was statistically longer than that for those without pancreatic metastases (27.9 months; 95% CI, 11.3–35.7; IQR, 12.4–35.7 vs. 7.0 months; 95% CI, 5.4–8.3; IQR, 2.9–16.6; *P* = 0.02). However, there was no difference in PFS for patients with and without pancreatic metastasis on atezolizumab (5.4 months; 95% CI, 2.6–8.5; IQR, 2.7–8.4 vs. 5.5 months; 95% CI, 3.0–8.4; IQR, 2.7–25.2) or on atezolizumab/bevacizumab (11.3 months; 95% CI, 1.2–not estimable; IQR, 1.2–31.9 vs. 11.0 months; 95% CI, 8.1–16.6; IQR, 5–25.3). In contrast, patients with pleural metastases had a worse PFS when treated with sunitinib compared with those without metastatic disease in the pleura (HR, 4.64; 95% CI, 1.92–11.17; *P* < 0.001). Kaplan-Meier analysis revealed a longer median PFS for patients with angiogenic cluster tumors (clusters 1 and 2) than those with other molecular clusters (clusters 3–7) on atezolizumab (median survival 8.31 [95% CI, 2.95–not estimable] vs. 2.99 [95% CI, 2.79–5.46] months, *P* = 0.007) and sunitinib (median survival 19.23 [95% CI, 11.28–27.88] vs. 5.42 [95% CI, 3.48–6.38] months, *P* < 0.001) (Figure 4). Although the median PFS of patients with angiogenic clusters on atezolizumab/bevacizumab was longer than that for other molecular clusters, it did not reach statistical significance (12.03 [95% CI, 8.55–27.06] vs. 5.92 [95% CI, 5.03–9.14] months, *P* = 0.06).

The presence of metastatic disease in the muscles or soft tissue as well as greater tumor count and greater tumor burden in these locations were associated with a worse PFS overall, predominantly driven by the atezolizumab treatment arm (Supplemental Table 6). Moreover, a worse PFS was observed (aggregating over all treatment arms) in patients with a greater tumor count or tumor burden in the lungs and lymph nodes. Finally, patients with a worse tumor burden in the retroperitoneum at baseline had worse PFS when treated with atezolizumab monotherapy (HR, 1.34; 95% CI, 1.14–1.57; *P* < 0.001).

## Discussion

We conducted a comprehensive analysis of over 5,000 metastatic sites in patients with metastatic RCC enrolled in the phase II IMmotion150 clinical trial and discovered that tumors in different molecular clusters differ in the anatomic distribution of metastases. We show a statistically higher prevalence of metastatic disease to the pancreas in patients with angiogenic ccRCCs (i.e., molecular signatures corresponding to clusters 1 and 2). Patients with pancreatic metastases were more likely to exhibit complete or partial response with sunitinib (OR, 7.13; 95% CI, 1.81–28.07, *P* = 0.0049). Notably, Kaplan-Meier analysis revealed that patients with pancreatic metastases exhibited an improved PFS rate when treated with the antiangiogenic drug sunitinib (*P* = 0.02). However, a similar benefit was not seen for patients with pancreatic metastases in the atezolizumab or atezolizumab/bevacizumab arms. Our observations are consistent with a higher overall response rate to the AA sunitinib observed in clusters 1 and 2 in the phase III IMmotion151 clinical trial, while other molecular clusters exhibited lower response to sunitinib (12, 13).

This study expands on our prior observations indicating that RCC tumors metastasizing to the pancreas are characterized by indolent biology (i.e., frequent *PBRM1* mutations, 3p loss, and 5q amplification, but a substantially lower frequency of *BAP1* mutations and loss of 9p, 14q, and 4q) (18, 19). These tumors are also characterized by heightened angiogenesis and an uninflamed stroma (18), phenotypic characteristics shared with RCC patients developing late-onset metastatic disease (19, 20). Such histologic and molecular characteristics are also consistent with their favorable prognosis, sensitivity to antiangiogenic therapies, and refractoriness to immune checkpoint inhibitors (18). In our study using a clinical trial patient population, rates of progression for patients with pancreatic metastases treated with sunitinib were lower than for those without pancreatic metastases (HR for progression, 0.52; 95% CI, 0.27–1.01; *P* = 0.052). There was an improved median PFS for patients with pancreatic metastases treated with sunitinib compared with those without (27.9 [95% CI, 11.3–35.7] months versus 7.0 [95% CI, 5.4–8.3] months; *P* = 0.02). In contrast, median PFS for patients treated with atezolizumab (with or without bevacizumab) was no different between patients with and without pancreatic metastases. Overall, these data suggest that patients with ccRCC with pancreatic metastases preferentially respond to TKIs. This is supported by recent studies using scRNA-seq analysis of tissue samples from pancreatic RCC metastases revealing the endothelial cell as the most active component of the tumor microenvironment (21). Importantly, pancreatic RCC metastases were sensitive to most TKIs, including sunitinib (21).

Involvement of other anatomic locations was also associated with differences in outcomes. A higher number of metastatic lymph nodes was associated with a worse response to therapy regardless of the treatment arm. Patients from clusters 1 and 2 had a lower number of lymph node metastases compared with clusters 4 and 5 (*P* = 0.0063) or compared with the remaining clusters (clusters 3, 6, and 7; *P* = 0.0094). Similarly, patients with bone metastases had a worse response independent of the treatment arm. This is consistent with prior reports of an overall worse prognosis for patients with metastatic RCC to the bones treated with molecular targeted therapies (22, 23) or the combination of ipilimumab/nivolumab (24). Ruatta et al. found that the number of bone metastases and concomitant metastases in other organs were associated with worse PFS and OS in metastatic RCC (22). Conversely, patients with solitary, single-bone metastasis had a better OS than those with multiple bone metastases. Unfortunately, the low frequency of patients (*n* = 4) exhibiting bone metastases as their only metastatic site in our study prevented further analysis. Santoni et al. reported that ECOG Performance Status, MSKCC group, and concomitant lung or lymph node metastases were independent predictors of overall survival in patients with bone metastases (23). We did not find such associations in the IMmotion150 cohort.

The current assessment of tumor response in patients with metastatic RCC relies on the Response Evaluation Criteria in Solid Tumors (RECIST) 1.1 guidelines, which include a maximum of 5 target lesions with no more than 2 from any given organ (25). While analyzing the total tumor burden would be impractical in clinical practice, our study offers some additional observations. First, although the lungs are the most common location of metastatic disease and harbor the largest number of metastases per organ, they do not represent the largest contributor to the total tumor burden. The explanation for this phenomenon is unclear. One possible explanation is that the lung architecture (or vasculature) cannot support large metastasis. Alternatively, a larger tumor burden in the lung may not be compatible with survival. Second, although the frequency and number of hepatic metastases are much lower (than that for lung and lymph node metastases), when present, they were the largest contributors to the total tumor burden in the molecular clusters 1 (angiogenic/stromal) and 3 (complement/ω-oxidation). Third, although the distribution of metastasis at baseline and first follow-up imaging was overall similar for most anatomic locations (Figure 1), brain metastases did not seem to occur at the same rate. However, this was influenced by lack of brain imaging at baseline or use of CT at baseline followed by MRI (i.e., more sensitive tests). Future research should assess the usefulness of these data for management decisions in both systemic and locoregional therapies (e.g., surgery, ablation, or stereotactic body radiation).

This study has some limitations. First, the generalizability of our results is limited by our relatively small sample size with very low instances of metastatic disease to some organs. An assessment of the total tumor burden in a larger cohort of patients with metastatic kidney cancer may enable better discrimination of anatomic phenotypes and their response to different therapies. Nevertheless, this is offset by the evaluation of patients in a large, prospective, interventional, phase II clinical trial. Second, out of the 3 therapeutic regiments in the IMmotion150 trial, only sunitinib is used in clinical practice and generally is no longer used as a first line therapy. Arguably, however, our data suggest that sunitinib may be a reasonable treatment option for patients with pancreatic metastases. Third, baseline biomarker analysis of transcriptome data was available in only 257/305 patients. Furthermore, the source of tissue samples was heterogeneous with 55.1% of the samples obtained from different metastatic sites, 42.2% from primary tumors, and 2.6% from unknown sites. Since tissue pairs of metastatic sites and primary tumor for a given patient were not obtained, it is not possible to assess the concordance of molecular clusters between primary tumors and metastases. However, analysis of the tumor burden in the entire cohort provided information about anatomic distribution of disease at baseline and response to treatment for each cluster. Data related to renal masses were excluded because of the heterogeneous presentation at enrollment (i.e., nephrectomy vs. primary tumor in situ) and inability to differentiate primary tumors from metastatic disease. Inclusion of primary tumors would have skewed the tumor volume data due to their generally larger size.

In summary, we performed a comprehensive analysis of the total tumor burden in patients with metastatic RCC from the IMmotion150 clinical trial. We found that the distribution of RCC metastases in the body is not random but rather related to the particular molecular cluster. Angiogenic tumors (molecular clusters 1 and 2) had a higher frequency and number of pancreatic metastases and lower number of lymph node metastases compared with other molecular clusters. In contrast, proliferative tumors (molecular clusters 4 and 5) had a higher number of lymph node metastases compared with other molecular clusters. Our data expand on prior reports on the response and survival rate for patients with pancreatic metastases receiving sunitinib therapy and their preferential response to TKI- versus IO-containing regimens. Previously defined molecular clusters that predict response to therapy are partially associated with tumor tropism to specific organs, such as the pancreas, and therefore can be recognized noninvasively with imaging. Radiologic studies offer a unique opportunity for optimization of therapy in metastatic patients for whom transcriptome data may not be available.

## Methods

### Sex as a biological variable.

Both male and female subjects were included in this study. Sex was recorded for all subjects; however, the study was not powered to detect sex-specific differences, and analyses were therefore not stratified by sex.

### Study cohort.

IMmotion150 was a multicenter, randomized, open-label, phase II clinical trial (ClinicalTrials.gov NCT01984242) that evaluated the efficacy, safety, and tolerability of atezolizumab as monotherapy or in combination with bevacizumab versus sunitinib in patients with histologically confirmed, inoperable, locally advanced, or metastatic RCC with a clear-cell and/or sarcomatoid component who had not received prior systemic therapy. We reviewed all available imaging data, demographics (age and sex), pathology, molecular subtype, and clinical outcomes (best response by RECIST 1.1 and PFS) for 305 patients. Baseline and first follow-up scans were included in the image analysis (median follow-up time 98 days, IQR 89–112 days). All clinical, pathology, transcriptome analysis, and response data by RECIST 1.1 were extracted from the originally reported IMmotion150 data.

### Image analysis.

The process for data storage and preparation is detailed in the supplemental materials. Volumetric segmentation of metastases was performed using a semiautomatic segmentation tool (mint Lesion version 8.2, Mint Medical) by a radiology resident (postgraduate year 2) and reviewed by a radiologist (2 years of clinical experience). A second radiologist (23 years of experience) reviewed the images in cases where there was disagreement, and a final interpretation was made in consensus between both radiologists. Consensus read due to disagreement occurred in less than 5% of lesions. All readers were blinded to pathology data, molecular analysis, and response by RECIST 1.1. A region of interest (ROI) was manually drawn along the outer margins of any visible metastases. A lesion was tabulated as a metastasis if it measured 1 cm or more in long-axis in the following anatomic locations: muscle, subcutaneous fat, bone, heart, pleura, adrenal gland, pancreas, liver, kidneys, spleen, peritoneum, retroperitoneum, pelvis, thyroid, and inferior vena cava tumor thrombus not contiguous with a primary renal mass. Lung nodules larger than 8 mm were tabulated as metastases, although subcentimeter nodules showing no change in size on 2 consecutive CT scans were excluded. Lymph nodes in the neck, chest, and abdomen were included when measuring greater than 1 cm in short-axis, whereas inguinal and axillary lymph nodes were included when measuring greater than 1.5 cm in short-axis (26, 27). Benign-appearing lymph nodes (i.e., bean-shaped with preserved fatty hilum and thin cortex) were excluded regardless of their location and size (26, 27). Adrenal lesions measuring less than 10 Hounsfield units, benign-appearing liver lesions (e.g., cystic, hemangiomas), and splenic infarcts were excluded regardless of their size. Renal masses (i.e., presumed primary tumor, ipsilateral, and contralateral renal masses) were excluded to avoid biases related to patients having undergone surgical resection of the renal mass.

### Data storage and preparation.

The compressed DICOM data, which included CT and MRI scans, was transferred to our institution from Genentech via an AWS S3 bucket. A total of 12,257 files were used, comprising 6,639 for body CT, 3,981 for body MRI, 475 for brain CT, and 1,162 for brain MRI. Initially, the compressed DICOM files were unzipped to normal DICOM files. Preprocessing of the DICOM images was then carried out, including splitting merged series and adding missing DICOM header information such as slice location and instance number to ensure proper display in mint Lesion software. Subsequently, the DICOM images were transferred to our research PACS and pushed to the mint Lesion server using the Clinical Trial Processor application. Annotations were performed using the 3D annotation tool in mint Lesion, and mask files were saved in Nrrd format and manually copied to a local high-performing computer cluster (BioHPC; https://www.utsouthwestern.edu/research/research-support/biohpc/). Images that could not be annotated using mint Lesion were annotated using the open-source software 3D Slicer (https://www.slicer.org/). Corrections for gantry tilt inconsistencies were made for certain brain images using acquisition geometry regularization, followed by annotation. All masks were saved in Nrrd format in 3D Slicer and transferred to bioHPC for final processing. Finally, all images were converted to NIfTI format using the DICOM-to-NIfTI conversion tool dcm2nii or 3D Slicer, depending on whether CT gantry tilting was present. Python was used with the SimpleITK module to compute or extract tumor volumes and related information from images and masks.

All CT images were classified based on their reconstruction settings (soft tissue, lung filter, and bone filter), anatomic area (head, neck, chest, abdomen, pelvis, or any combination of them), contrast enhancement (unenhanced vs. contrast enhanced), and contrast phase (arterial, venous, and delayed).

### Statistics.

All metastatic sites were classified into 1 of 14 organs: adrenal gland, bone, lung, liver, lymph node, muscle/soft tissue, CNS, pancreas, heart, peritoneum, pleura, spleen, thyroid, and retroperitoneum. For each organ, the acquisition(s) exhibiting the greatest number of metastases was identified from all available acquisitions (i.e., different reconstruction settings, anatomic area, contrast enhancement, and phase) and recorded as the total number of lesions. The corresponding maximum total tumor burden defined as the sum of the volume (cm^3^) of these metastases was calculated.

The lack of ROI reported in an organ could be due to the lack of imaging data (e.g., absence of chest CT) or to the lack of metastatic disease in that organ (e.g., absence of visible metastasis on chest CT). To resolve this, all 14 organs were further categorized into 4 groups: CNS, chest only organ (heart, lung, pleura, and thyroid), abdomen/pelvis only organ (adrenal gland, liver, pancreas, peritoneum, retroperitoneum, and spleen), and either (bone, muscle/soft tissue, and lymph nodes), based on their anatomical locations. An organ was marked as free of metastatic disease only when no ROI was placed and the organ was visible in at least 1 of the available image datasets. For bone, muscle/soft tissue, and lymph nodes, chest, abdomen, and pelvis datasets were required. Five patients in whom only chest and abdomen imaging was available (i.e., no pelvis) without visible metastases in the bones or soft tissue, were tabulated as negative for the presence of osseous or soft-tissue metastases. Metastatic disease in the heart was only found in 2 patients and therefore was removed from all organ-specific analysis.

The binary indicator of metastasis (present versus absent), total tumor count (total number of metastases), and total tumor burden (volumetric [cm^3^] sum of all metastases) for each organ per patient were tabulated for both baseline and first follow-up scans. Radar plots were generated to illustrate the distribution across organs.

The associations between the organ-specific metastatic distribution and molecular subtypes (i.e., angiogenic [clusters 1 and 2] vs. the rest and proliferative [clusters 4 and 5] vs. the rest) were assessed using Pearson’s χ^2^ tests for disease presence and Wilcoxon’s rank sum tests for total tumor count and burden.

Multivariable logistic regression models were applied with the best response according to RECIST 1.1 as the dependent variable and the treatment arm and metastasis distribution [binary indicator, log_2_ (total tumor count + 1) or log_2_ (total tumor burden + 1)] as the independent variables for each organ. Two types of logistic regression models were applied. First, with metastasis distribution and treatment arms as 2 main effects; second, with additional interaction term between metastasis distribution and treatment arms. The first model provided overall estimates of the effect of metastasis distribution (i.e., the main metastasis distribution effect as the balanced average across all treatment arms), and the second model allowed estimates of the effect of metastasis distribution conditional on treatment arm. Corresponding ORs for the metastasis distribution were reported. OR for total tumor count/burden with log_2_ transformation can be interpreted as ratio of odds (i.e., of complete response/partial response [CR/PR] against stable disease/progressive disease [SD/PD]) for every doubling of total tumor count/burden. Given that the primary interest was estimation of treatment- and disease-specific ORs rather than confirmatory hypothesis testing, no adjustment for multiple comparisons was applied in this part of the analysis, as such corrections would be overly conservative and substantially reduce power to detect disease-specific associations.

Similar analyses were done using Cox proportional hazard regression models with PFS as the dependent variable. Corresponding HRs were reported. HR for total tumor count/burden with log_2_ transformation can be interpreted as ratio of HR for every doubling of total tumor count/burden. Kaplan-Meier analysis was also used when appropriate.

The details of the transcriptomic analysis completed in this work have been previously reported (13). Briefly, RNA was extracted from FFPE tissue using the High Pure FFPET RNA Isolation Kit (Roche). Whole-transcriptome profiles were generated using TruSeq RNA Access technology (Illumina). The 7 transcriptionally defined subgroups of RCC were defined using a non-negative matrix factorization (NMF) method in IMmotion151 and validated in the IMmotion150 cohort (13). NMF is an unsupervised clustering algorithm that iteratively selects the most robust clustering pattern within a given dataset. No additional analysis was performed beyond what was reported by Motzer et al. (13). The previously reported cluster assignment for each patient was incorporated into the analysis in this work, without further transcriptomic analysis.

All patients were categorized into 3 groups according to their corresponding molecular subtypes (1 and 2 vs. 4 and 5 vs. the rest). Logistic regression models were used to assess the association between molecular subtypes and the best response according to RECIST 1.1 adjusted for treatment arm. Corresponding ORs were reported with Holm’s adjusted *P* values for pairwise comparisons. The significance level was set at 0.05. All analyses were done in SAS 9.4 (SAS Institute) and R 4.5.1 (R Core Team).

### Data availability.

The data that support the findings of the IMmotion150 study, including anonymized genetic data from 163 patients who granted informed consent to share such data, were made available at the European Genome-Phenome Archive under accession number EGAS00001002928 after publication of the study results (11). Furthermore, qualified researchers have access to additional individual clinical patient level data through the clinical study data request platform (https://clinicalstudydatarequest.com/). Details of Roche’s Data Sharing Policy are available here: https://www.roche.com/innovation/clinical-trials/data-sharing Volumetric data of tumor segmentations used in this study may be made available to qualified researchers upon request. Values for all data points in figures are provided in the Supporting Data Values file, with each figure panel in a separate tab.

## Author contributions

GH, JG, and YX conducted experiments, curated data, performed formal analyses, developed methodology, validated results, generated visualizations, and wrote the original draft of the manuscript. JG and YX developed software and computational pipelines used in the analysis. EA curated data and contributed to formal analyses. MH, HH, RB, and EK curated data and reviewed and edited the manuscript. SD and CC contributed to data interpretation and reviewed and edited the manuscript. PK and JB contributed to study conceptualization and reviewed and edited the manuscript. IP conceived and supervised the study, designed the research methodology, curated and analyzed data, acquired funding and resources, administered the project, validated results, generated visualizations, and wrote and revised the manuscript. All authors reviewed and approved the final version of the manuscript.

## Conflict of interest

IP, YX, JG, EA, JB, and PK received salary support from the NIH. IP serves as advisor and has received warrants from Health Tech International. PK received funding from the Department of Defense and Cancer Prevention and Research Institute of Texas. JB reports grants from Bristol Myers Squibb, Genentech, Telix, and ImaginAb; consulting fees from Regeneron; an active license with Merck and Bethyl; and multiple issued and pending patents (patents 12,234,516; 18/965,965; 11,576,889; 12,161,618; 17/153,128; 17/129,352; 19/083,289; and 63/766,832). CC, HH, SD, EK, RB, and MH are Genentech employees or were at the time of analysis. CC, SD, RB, and MH hold Genentech stock. EK holds or has held Apple, Allogene, Amazon, Annovis, AstraZeneca, 89bio, Google, Eli Lilly, Merck, Nvidia, Roche, Revolution Medicines, Teledoc, Tempus, and State Street SPDR S&P Biotech ETF stock. EK is an inventor on US patent 11,254,987 B2.

## Funding support

This work is the result of NIH funding, in whole or in part, and is subject to the NIH Public Access Policy. Through acceptance of this federal funding, the NIH has been given a right to make the work publicly available in PubMed Central.

- NIH grant R01CA154475 (to GH, JG, YX, PK, and IP).
- NIH grant P50CA196516 (to PK, JB, and IP).

## Supplementary Material

Supplemental data

ICMJE disclosure forms

Supporting data values

## Figures and Tables

**Table 1:**
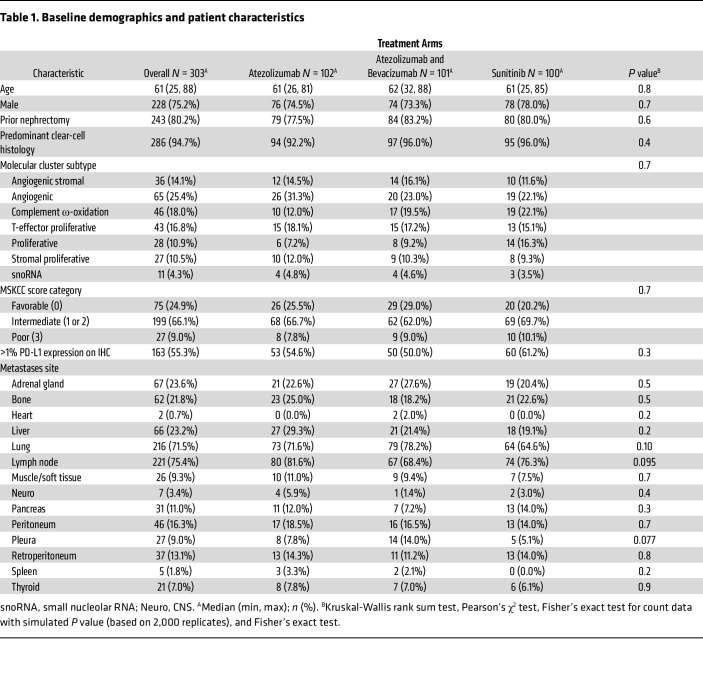
Baseline demographics and patient characteristics

**Table 2:**
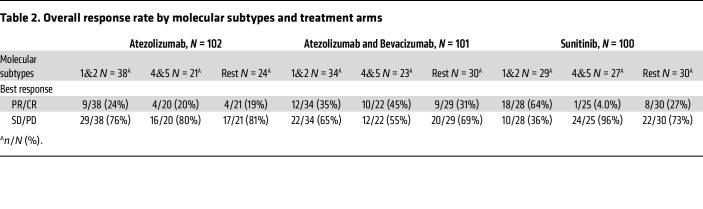
Overall response rate by molecular subtypes and treatment arms

## Version 1 05/15/2026

Electronic publication
