# Supplementary material for: Metastatic tropism of molecularly defined clear-cell renal cell carcinoma clusters
Source: J Clin Invest. 2026 May 15;136(10):e195288. doi: 10.1172/JCI195288 (PMC13178644; doi:10.1172/JCI195288)
Supplement: Supplemental data [file jci-136-195288-s073.pdf]

Supplementary material:

Supp. Table 1: Baseline and follow up CT and MRI scans of different anatomic regions, including different contrast enhancement phases of CT examinations.

Baseline studies							
Anatomic Region	Modality	Total number of studies	Non-enhanced	Enhanced	Arterial phase	Venous phase	Delayed phase
Brain	CT	201	71	130	..	..	..
Brain	MRI	64	0	64	..	..	..
Neck	CT	18	4	10	1	13	..
Chest	CT	530	86	444	105	165	2
Abdomen	CT	426	76	350	74	241	33
Pelvis	CT	314	37	277	19	235	21
Follow-up studies							
Anatomic Region	Modality	Total number of studies	Non-enhanced	Enhanced			
Brain	CT	52	..	52	..	..	..
Brain	MRI	29	..	29	..	..	..
Neck	CT	14	1	13	1	12	0
Chest	CT	391	60	331	29	134	1
Abdomen	CT	388	73	315	65	222	28
Pelvis	CT	297	40	257	20	217	20

CT: computer tomography, MRI: magnetic resonance imaging.

Supp. Table 2 : Sites of tissue sampling.

**Characteristic**	**N = 303**
Primary/Metastasis	
Primary	128 (42.2%)
Metastasis	167 (55.1%)
Unknown/Not collected	8 (2.6%)
Sample Location	
Kidney	132 (43.6%)
Lung	60 (19.8%)
Bone	26 (8.6%)
Unknown/Not collected	17 (5.6%)
Liver	13 (4.3%)
Muscle/soft tissue	12 (4.0%)
Lymph node	8 (2.6%)
Retroperitoneum	7 (2.3%)
Adrenal	6 (2.0%)
Extremities	4 (1.3%)
Pancreas	4 (1.3%)
Pleura	4 (1.3%)
Abdomen (non-specific, not kidney)	1 (0.3%)
Bowel	1 (0.3%)
Left back mass	1 (0.3%)
Neuro	1 (0.3%)
Oral mucosa	1 (0.3%)
Right hilar mass	1 (0.3%)
Spleen	1 (0.3%)
Testis	1 (0.3%)
Thyroid	1 (0.3%)
Vagina	1 (0.3%)
Collection Type	
Biopsy	159 (52.5%)
Surgery	106 (35.0%)
Unknown/Not collected	37 (12.2%)
Bronchoscopy	1 (0.3%)

Supp. Table 3: Comparison of the characteristics of pancreatic metastases and the number of lymph nodes between clusters one and two and the rest of the clusters, and clusters four and five and the rest of the clusters.

	Clusters 1 and 2	Other clusters	P-value
Pancreatic metastases	18 (18.9%)	8 (5.6%)	0.001
Number of pancreatic metastases	0.60 ± 1.88	0.07 ± 0.31	<0.001
Pancreas metastatic burden (cm^3)	4.2 ± 13.7	0.7 ± 3.4	0.001
Number of lymph nodes	2.5 ± 3.7	4.1 ± 5.6	0.010
	Clusters 4 and 5	Other clusters	P-value
Number of lymph nodes	5.5 ± 7.2	2.7 ± 3.6	0.012

Supp. Table 4: Effect of the presence of disease, total tumor burden, and total tumor count in different organs on best response aggregated and stratified by treatment arms.

	Organ	Total Cohort	False Discovery Rate p-value	Atezolizumab	Atezolizumab plus Bevacizumab	Sunitinib
Presence of Disease	CNS	2.23 (0.43, 11.58) p = 0.3	0.3	0.93 (0.1, 8.48) p > 0.9	6.6 (0.07, 608.27) p = 0.4	5.62 (0.06, 518.06) p = 0.5
	adrenal gland	1 (0.55, 1.82) p > 0.9	>0.9	1.33 (0.45, 3.97) p = 0.6	1.41 (0.56, 3.53) p = 0.5	0.49 (0.15, 1.59) p = 0.2
	bone	0.44 (0.22, 0.89) p = 0.02	0.043	0.69 (0.21, 2.26) p = 0.5	0.11 (0.02, 0.66) p = 0.02	0.83 (0.29, 2.43) p = 0.7
	liver	0.8 (0.42, 1.49) p = 0.5	0.5	1.2 (0.42, 3.38) p = 0.7	0.27 (0.08, 0.97) p = 0.044	1.53 (0.52, 4.53) p = 0.4
	lung	1.25 (0.71, 2.2) p = 0.4	0.4	0.88 (0.32, 2.41) p = 0.8	1.54 (0.55, 4.35) p = 0.4	1.33 (0.54, 3.27) p = 0.5
	lymph node	1.11 (0.61, 1.99) p = 0.7	0.7	1.4 (0.38, 5.15) p = 0.6	1.59 (0.64, 3.97) p = 0.3	0.6 (0.23, 1.57) p = 0.3
	muscle/ soft tissue	1.22 (0.49, 3.01) p = 0.7	0.7	0.14 (0.01, 2.94) p = 0.2	3.56 (0.84, 15.03) p = 0.08	1.42 (0.23, 8.87) p = 0.7
	pancreas	1.37 (0.62, 3.04) p = 0.4	0.4	0.11 (0.01, 2.29) p = 0.2	0.75 (0.14, 3.94) p = 0.7	7.13 (1.81, 28.07) p = 0.005
	peritoneum	1.64 (0.84, 3.19) p = 0.1	0.1	2.12 (0.67, 6.69) p = 0.2	1.43 (0.48, 4.24) p = 0.5	1.51 (0.44, 5.22) p = 0.5
	pleura	1.68 (0.67, 4.23) p = 0.3	0.3	0.81 (0.11, 6) p = 0.8	3.29 (0.99, 10.95) p = 0.05	0.38 (0.01, 15.98) p = 0.6
	retroperitoneum	0.97 (0.44, 2.13) p > 0.9	>0.9	1.3 (0.32, 5.29) p = 0.7	0.78 (0.19, 3.17) p = 0.7	1.03 (0.29, 3.7) p > 0.9
	spleen	0.96 (0.11, 8.5) p > 0.9	>0.9	3.2 (0.19, 53.41) p = 0.4	0.33 (0.01, 13.9) p = 0.6	
	thyroid	1.91 (0.76, 4.82) p = 0.2	0.2	4.48 (0.93, 21.61) p = 0.06	0.79 (0.15, 4.15) p = 0.8	2.05 (0.39, 10.79) p = 0.4
Total Tumor Count	CNS	1.75 (0.4, 7.67) p =0.5	0.5	0.87 (0.13, 5.89) p = 0.9	6.6 (0.07, 608.27) p = 0.4	5.62 (0.06, 518.06) p = 0.5
	adrenal gland	0.95 (0.58, 1.55) p = 0.8	0.8	1.39 (0.51, 3.8) p = 0.5	1.11 (0.56, 2.2) p = 0.8	0.55 (0.2, 1.52) p = 0.3
	bone	0.54 (0.34, 0.87) p = 0.01	0.02	0.7 (0.33, 1.49) p = 0.4	0.18 (0.04, 0.84) p = 0.03	0.77 (0.43, 1.35) p = 0.4
	liver	0.93 (0.69, 1.24) p = 0.6	0.6	1.11 (0.72, 1.71) p = 0.6	0.5 (0.24, 1.06) p = 0.07	1.23 (0.74, 2.04) p = 0.4
	lung	0.99 (0.86, 1.13) p = 0.8	0.8	0.9 (0.69, 1.18) p = 0.5	1.04 (0.82, 1.33) p = 0.7	1.01 (0.81, 1.25) p > 0.9
	lymph node	0.91 (0.74, 1.12) p = 0.4	0.4	1.08 (0.7, 1.67) p = 0.7	1.07 (0.78, 1.47) p = 0.7	0.67 (0.46, 0.98) p = 0.04
	muscle/soft tissue	1.12 (0.67, 1.88) p = 0.7	0.7	0.17 (0.01, 2.93) p = 0.2	1.43 (0.7, 2.94) p = 0.3	1.19 (0.46, 3.11) p = 0.7
	pancreas	1.15 (0.72, 1.83) p = 0.6	0.6	0.23 (0.03, 1.94) p = 0.2	0.74 (0.23, 2.36) p = 0.6	2.37 (1.03, 5.47) p = 0.043
	peritoneum	1.27 (0.9, 1.78) p = 0.2	0.2	2.08 (0.94, 4.61) p = 0.07	1.2 (0.74, 1.94) p = 0.5	1.08 (0.59, 1.96) p = 0.8
	pleura	1.09 (0.76, 1.58) p = 0.6	0.6	0.89 (0.45, 1.74) p = 0.7	1.57 (0.88, 2.78) p = 0.1	0.67 (0.09, 5.02) p = 0.7
	retroperitoneum	0.97 (0.65, 1.47) p = 0.9	0.9	1 (0.34, 2.94) p > 0.9	0.98 (0.47, 2.06) p > 0.9	1.01 (0.59, 1.74) p > 0.9
	spleen	1.37 (0.24, 7.96) p = 0.7	0.7	2.87 (0.33, 25.19) p = 0.3	0.33 (0.01, 13.91) p = 0.6	

	thyroid	1.83 (0.9, 3.72) p = 0.10	0.1	3.13 (0.94, 10.37) p = 0.06	1.13 (0.3, 4.19) p = 0.9	1.52 (0.43, 5.33) p = 0.5
Total Tumor Burden	CNS	1.24 (0.72, 2.14) p = 0.4	0.4	1.06 (0.57, 1.95) p = 0.9	2.19 (0.33, 14.44) p = 0.4	3.02 (0.17, 54.71) p = 0.5
	adrenal gland	0.99 (0.85, 1.15) p > 0.9	>0.9	1.04 (0.8, 1.37) p = 0.7	1.06 (0.83, 1.34) p = 0.6	0.88 (0.66, 1.19) p = 0.4
	bone	0.82 (0.7, 0.95) p = 0.007	0.01	0.87 (0.69, 1.11) p = 0.3	0.6 (0.38, 0.96) p = 0.03	0.92 (0.75, 1.12) p = 0.4
	liver	0.98 (0.87, 1.1) p = 0.7	0.7	1.08 (0.9, 1.3) p = 0.4	0.82 (0.65, 1.04) p = 0.10	1.11 (0.86, 1.43) p = 0.4
	lung	1 (0.89, 1.13) p > 0.9	>0.9	0.92 (0.73, 1.17) p = 0.5	1.21 (0.97, 1.52) p = 0.09	0.92 (0.76, 1.13) p = 0.4
	lymph node	0.94 (0.84, 1.04) p = 0.2	0.2	1.03 (0.83, 1.27) p = 0.8	1 (0.85, 1.19) p > 0.9	0.82 (0.68, 0.99) p = 0.04
	muscle/soft tissue	1.07 (0.85, 1.34) p = 0.6	0.6	0.54 (0.18, 1.58) p = 0.3	1.3 (0.93, 1.81) p = 0.1	0.97 (0.55, 1.72) p > 0.9
	pancreas	1.04 (0.85, 1.28) p = 0.7	0.7	0.58 (0.25, 1.36) p = 0.2	0.79 (0.48, 1.3) p = 0.3	1.78 (1.18, 2.69) p = 0.006
	peritoneum	1.09 (0.93, 1.27) p = 0.3	0.3	1.26 (0.85, 1.87) p = 0.2	1.1 (0.9, 1.35) p = 0.4	1 (0.73, 1.37) p > 0.9
	pleura	1.1 (0.9, 1.35) p = 0.3	0.3	0.92 (0.57, 1.47) p = 0.7	1.28 (0.97, 1.68) p = 0.08	0.74 (0.18, 3.04) p = 0.7
	retroperitoneum	0.94 (0.79, 1.13) p = 0.5	0.5	0.87 (0.55, 1.39) p = 0.6	0.97 (0.72, 1.3) p = 0.8	0.99 (0.77, 1.27) p > 0.9
	spleen	0.76 (0.16, 3.71) p = 0.7	0.7	3.06 (0.16, 59.2) p = 0.5	0.56 (0.07, 4.41) p = 0.6	
	thyroid	1.16 (0.86, 1.56) p = 0.3	0.3	1.74 (1, 3.03) p = 0.0498	0.85 (0.44, 1.62) p = 0.6	1.06 (0.66, 1.69) p = 0.8

Supp Table 5: Effect of molecular subtypes on best response stratified by treatment arms.

Treatment arms	Clusters	Vs. Clusters	OR	adjusted p-value
Atezolizumab	1&2	4&5	1.18 (0.32, 4.33)	1
	1&2	rest	1.25 (0.34, 4.57)	1
	4&5	rest	1.06 (0.24, 4.78)	1
Atezolizumab plus Bevacizumab	1&2	4&5	0.66 (0.22, 1.97)	1
	1&2	rest	1.2 (0.42, 3.43)	1
	4&5	rest	1.81 (0.58, 5.71)	1
Sunitinib	1&2	4&5	28.78 (4.53, 182.99)	0.003
	1&2	rest	4.66 (1.53, 14.19)	0.053
	4&5	rest	0.16 (0.03, 1.04)	0.4

Supp Table 6: Effect of the presence of disease, total tumor burden, and total tumor count in different organs on progression free survival aggregated and stratified by treatment arms.

	Organ	Total Cohort	False Discovery Rate p-value	Atezolizumab	Atezolizumab plus Bevacizumab	Sunitinib
Presence of Disease	CNS	0.84 (0.33, 2.15) p =0.71	0.9	1.52 (0.51, 4.56) p =0.45	0.36 (0.02, 6.02) p =0.48	0.88 (0.17, 4.55) p =0.88
	adrenal gland	0.85 (0.62, 1.16) p =0.30	0.5	0.65 (0.36, 1.15) p =0.14	0.88 (0.53, 1.46) p =0.61	1.12 (0.65, 1.92) p =0.68
	bone	0.741 (0.74, 1.41) p =0.89	0.9	0.84 (0.49, 1.46) p =0.54	1.31 (0.75, 2.3) p =0.34	0.605 (0.61, 1.82) p =0.86
	liver	0.766 (0.77, 1.43) p =0.77	0.8	0.578 (0.58, 1.61) p =0.89	1.35 (0.79, 2.32) p =0.27	0.524 (0.52, 1.66) p =0.81
	lung	1.22 (0.911, 1.63) p =0.18	0.3	1.12 (0.68, 1.84) p =0.66	0.589 (0.59, 1.76) p =1.0	1.44 (0.903, 2.3) p =0.13
	lymph node	1.29 (0.937, 1.77) p =0.12	0.4	0.563 (0.56, 1.78) p =1.0	1.53 (0.9, 2.59) p =0.12	1.26 (0.74, 2.15) p =0.40
	muscle/ soft tissue	1.59 (1.033, 2.45) p =0.04	0.1	3.83 (1.95, 7.52) p =<0.001	0.482 (0.48, 2.21) p =0.9	1.28 (0.57, 2.89) p =0.55
	pancreas	0.82 (0.54, 1.26) p =0.37	0.6	1.86 (0.958, 3.61) p =0.07	0.84 (0.35, 2.02) p =0.69	0.52 (0.27, 1.006) p =0.05
	peritoneum	0.82 (0.56, 1.18) p =0.28	0.5	0.75 (0.4, 1.41) p =0.37	0.506 (0.51, 1.72) p =0.82	0.84 (0.43, 1.61) p =0.59
	pleura	1.5 (0.968, 2.34) p =0.07	0.2	1.85 (0.87, 3.96) p =0.11	0.555 (0.55, 1.96) p =0.90	4.64 (1.92, 11.17) p =<.001
	retroperitoneum	1.12 (0.76, 1.67) p =0.56	0.6	1.26 (0.65, 2.44) p =0.49	1.83 (0.947, 3.52) p =0.07	0.72 (0.35, 1.48) p =0.37
	spleen	1.55 (0.6, 4.01) p =0.37	0.6	0.29 (0.29, 3.7) p =1.0	4.63 (1.28, 16.73) p =0.02	
	thyroid	0.71 (0.43, 1.18) p =0.19	0.3	0.403 (0.4, 2.03) p =0.81	0.422 (0.42, 2.13) p =0.89	0.48 (0.18, 1.27) p =0.14
Total Tumor Count	CNS	0.391 (0.39, 2.16) p =0.85	0.9	1.54 (0.61, 3.88) p =0.36	0.36 (0.02, 6.02) p =0.48	0.88 (0.17, 4.54) p =0.88
	adrenal gland	0.89 (0.69, 1.16) p =0.39	0.6	0.62 (0.36, 1.071) p =0.09	0.648 (0.65, 1.4) p =0.81	1.16 (0.74, 1.84) p =0.52
	bone	1.17 (0.981, 1.38) p =0.08	0.2	1.21 (0.85, 1.72) p =0.29	1.42 (1.024, 1.96) p =0.04	0.828 (0.83, 1.38) p =0.61
	liver	1.11 (0.97, 1.27) p =0.13	0.4	0.867 (0.87, 1.3) p =0.56	1.32 (1.028, 1.69) p =0.03	0.818 (0.82, 1.39) p =0.63
	lung	1.11 (1.032, 1.19) p =0.005	0.01	0.971 (0.971, 1.24) p =0.13	0.949 (0.949, 1.26) p =0.22	1.14 (1.011, 1.28) p =0.03
	lymph node	1.16 (1.042, 1.29) p =0.007	0.02	1.13 (0.921, 1.38) p =0.24	1.18 (1, 1.4) p =0.05	1.16 (0.966, 1.4) p =0.11
	muscle/ soft tissue	1.26 (0.987, 1.6) p =0.06	0.2	2.89 (1.69, 4.92) p =<.001	1.16 (0.8, 1.68) p =0.44	1.11 (0.72, 1.72) p =0.64
	pancreas	0.88 (0.69, 1.13) p =0.33	0.5	1.37 (0.904, 2.09) p =0.14	0.82 (0.45, 1.51) p =0.53	0.76 (0.53, 1.1) p =0.15
	peritoneum	0.757 (0.76, 1.12) p =0.43	0.6	0.78 (0.51, 1.19) p =0.24	0.777 (0.78, 1.36) p =0.85	0.697 (0.7, 1.34) p =0.84
	pleura	1.2 (1.02, 1.42) p =0.03	0.08	1.31 (1.061, 1.62) p =0.01	0.764 (0.76, 1.33) p =1.0	2.17 (1.43, 3.27) p =<.001
	retroperitoneum	0.842 (0.84, 1.29) p =0.71	0.7	1.45 (0.902, 2.34) p =0.12	1.3 (0.914, 1.84) p =0.15	0.85 (0.6, 1.19) p =0.34
	spleen	1.19 (0.58, 2.44) p =0.63	0.6	0.391 (0.39, 2.3) p =0.9	4.64 (1.28, 16.74) p =0.02	
	thyroid	0.78 (0.53, 1.15) p =0.21	0.3	0.554 (0.55, 1.78) p =1.0	0.5 (0.5, 1.76) p =0.84	0.58 (0.28, 1.2) p =0.14
Total Tumor Burden	CNS	0.795 (0.79, 1.39) p =0.73	0.9	0.783 (0.78, 1.49) p =0.64	0.65 (0.2, 2.11) p =0.48	1.51 (1.02, 2.24) p =0.04
	adrenal gland	0.89 (0.89, 1.045) p =0.38	0.6	0.88 (0.76, 1.026) p =0.10	0.86 (0.86, 1.13) p =0.83	0.927 (0.927, 1.2) p =0.42
	bone	0.96 (0.964, 1.081) p =0.48	0.6	0.907 (0.907, 1.097) p =1.0	0.99 (0.99, 1.21) p =0.08	0.909 (0.909, 1.11) p =1.0

	liver	0.954 (0.954, 1.07) p =0.73	0.7	0.869 (0.87, 1.046) p =0.31	0.986 (0.986, 1.18) p =0.10	0.924 (0.924, 1.2) p =0.44
	lung	1.033 (1.033, 1.17) p =0.003	0.009	1.1 (0.992, 1.22) p =0.07	0.891 (0.89, 1.13) p =0.9	1.18 (1.066, 1.31) p =0.001
	lymph node	1.022 (1.022, 1.14) p =0.006	0.02	0.962 (0.962, 1.18) p =0.23	0.996 (0.996, 1.19) p =0.06	0.988 (0.988, 1.19) p =0.09
	muscle/ soft tissue	1.12 (1.005, 1.25) p =0.041	0.1	1.4 (1.17, 1.69) p =<.001	0.846 (0.85, 1.19) p =1.0	1.23 (0.966, 1.56) p =0.09
	pancreas	0.85 (0.85, 1.064) p =0.38	0.6	1.12 (0.952, 1.31) p =0.18	0.82 (0.82, 1.27) p =0.84	0.83 (0.69, 0.998) p =0.048
	peritoneum	0.899 (0.9, 1.072) p =0.68	0.7	0.758 (0.76, 1.18) p =0.61	0.894 (0.89, 1.12) p =1.0	0.845 (0.84, 1.17) p =1.0
	pleura	1.13 (1.041, 1.23) p =0.004	0.01	1.27 (1.12, 1.43) p =<.001	0.906 (0.906, 1.15) p =0.72	1.65 (1.35, 2) p =<.001
	retroperitoneum	0.98 (0.98, 1.16) p =0.14	0.4	1.34 (1.14, 1.57) p =<.001	1.16 (1.004, 1.34) p =0.044	0.811 (0.81, 1.09) p =0.42
	spleen	1.92 (1.31, 2.81) p =<.001	0.003	1.93 (1.25, 2.99) p =0.003	2.19 (1.12, 4.29) p =0.02	
	thyroid	0.78 (0.78, 1.072) p =0.28	0.4	0.755 (0.76, 1.28) p =0.89	0.759 (0.76, 1.35) p =0.9	0.86 (0.66, 1.12) p =0.25

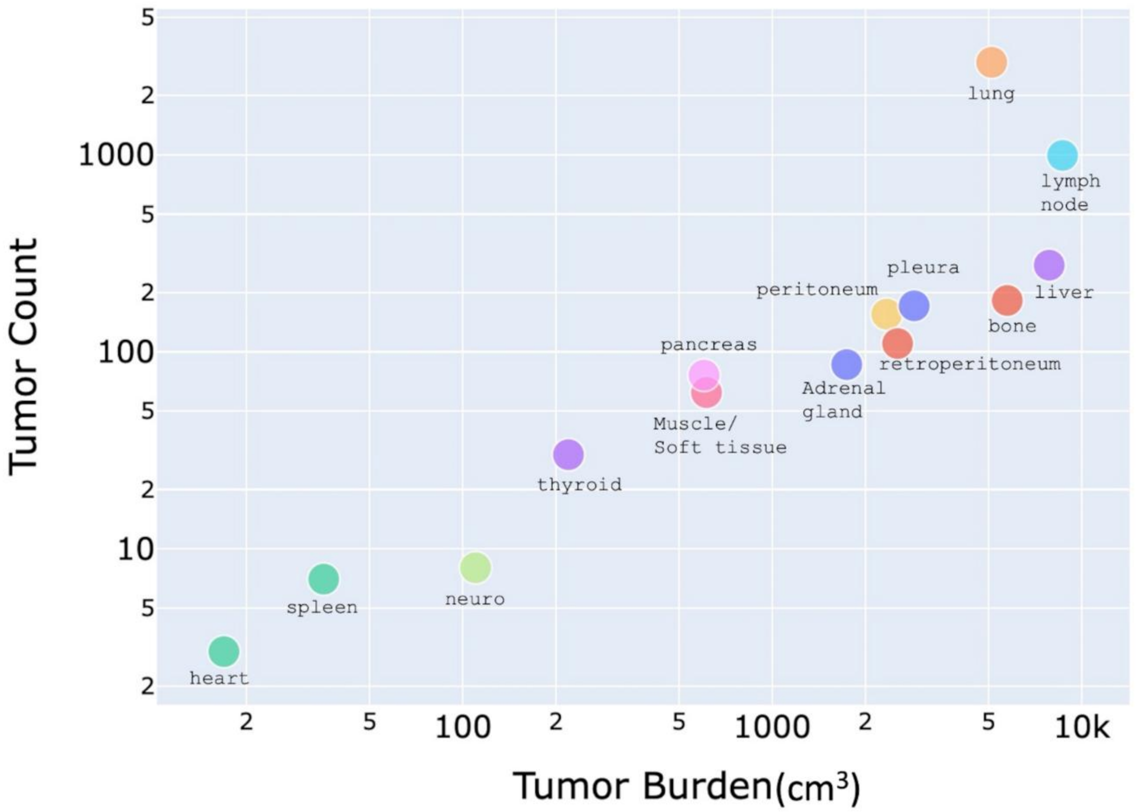

Supp. Fig. 1: Relationship between number of metastases and tumor burden by anatomic location. Scatter plot representing the total tumor count (i.e., number of metastasis) vs. the total tumor burden (cm^3) by anatomic location in the entire cohort at baseline.

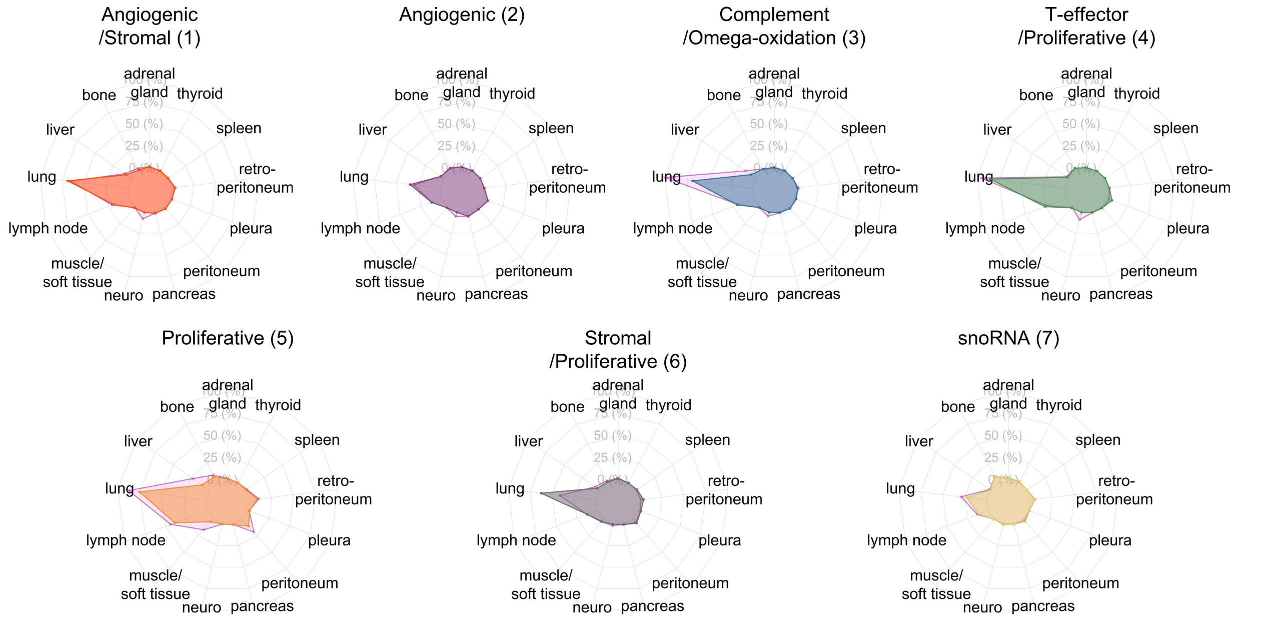

Supp. Fig. 2: Radar plot of the average number of metastases in each anatomic location. Each graph displays the tumor count on an exponential scale in each anatomic location. The colored line of each diagram corresponds to the baseline imaging data, and the grey line to the data at the time of the first imaging follow up.

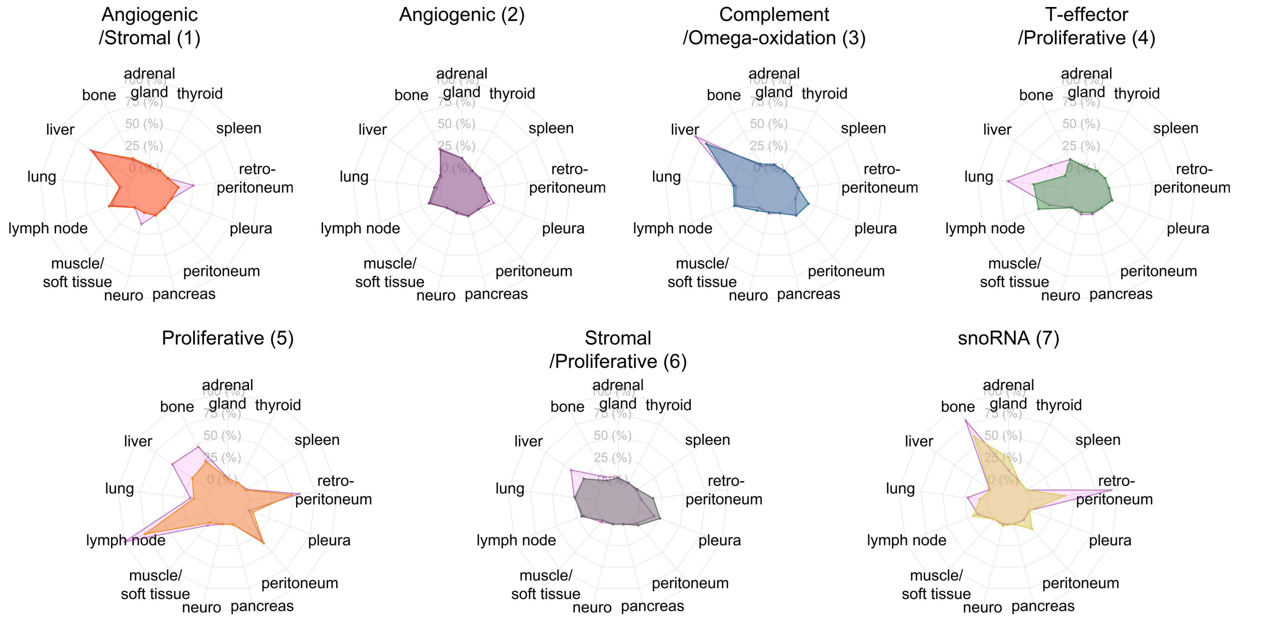

Supp. Fig. 3: Radar plot of the total tumor burden (cm^3) at each anatomic location. Each graph displays the tumor burden on an exponential scale in each anatomic location. The colored line of each diagram corresponds to the baseline imaging data, and the grey line to the data at the time of the first imaging follow up.

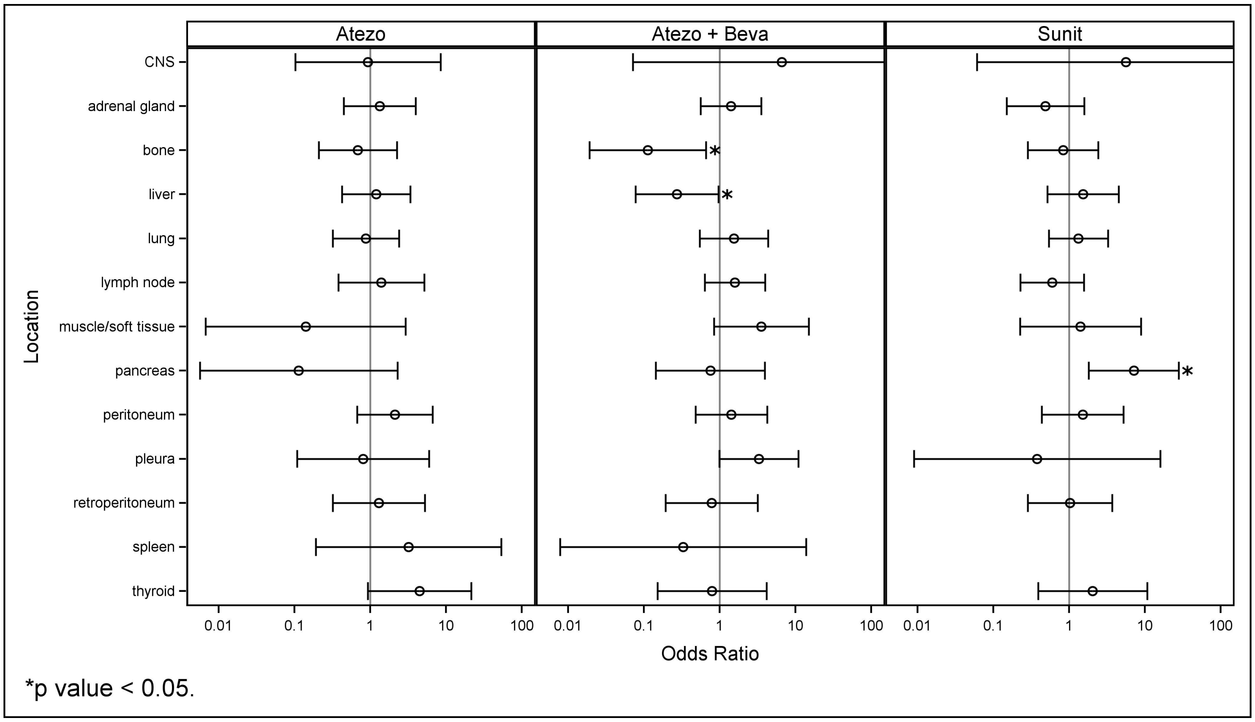

Supp. Fig. 4: Response to treatment according to the presence of metastases in different organs in the three different treatment arms. Estimated odds ratios (circles) and 95% confidence intervals (brackets) for best response (CR/PR vs. SD/PD) based on the presence of metastatic disease in each anatomic location for each treatment arm. Higher odds represent higher likelihood of response. Atezo = atezolizumab arm; Atezo + Beva = atezolizumab plus bevacizumab arm.

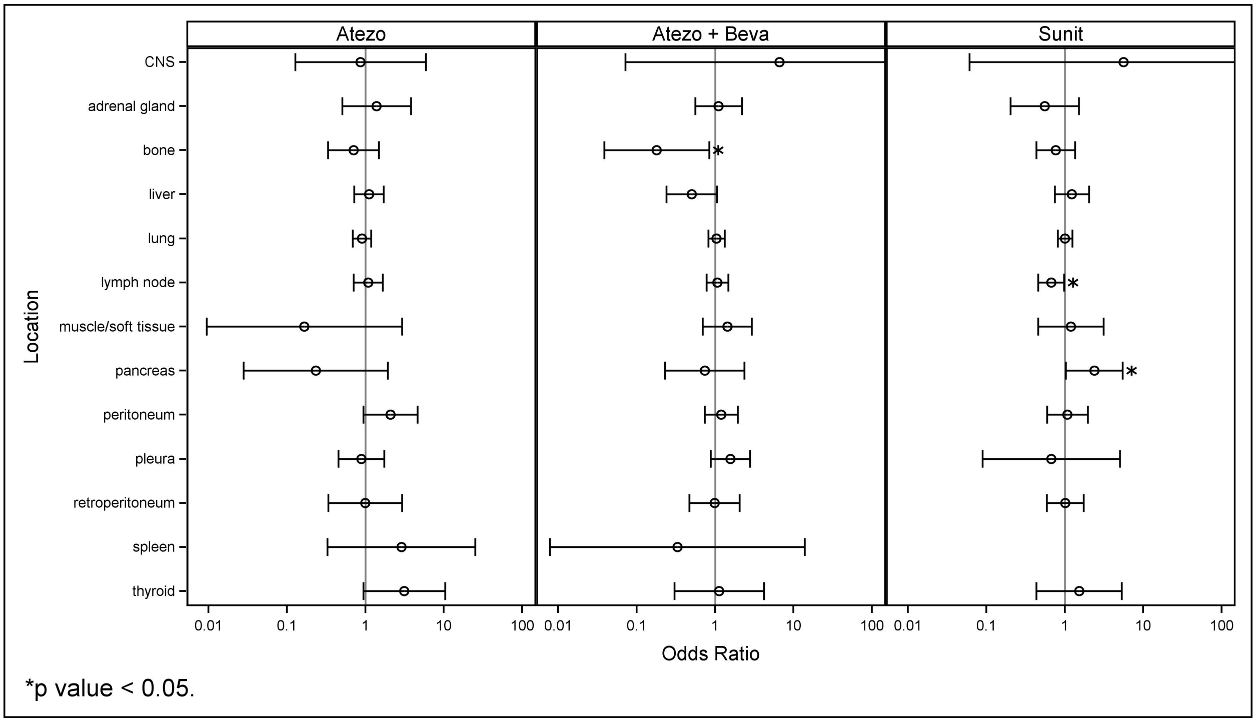

Supp. Fig. 5: Response to treatment according to the tumor count in different organs in the three different treatment arms. Estimated odds ratios (circles) and 95% confidence intervals (brackets) for best response (CR/PR vs. SD/PD) based on the number of metastases in each anatomic location for each treatment arm. Higher odds represent higher likelihood of response. Atezo = atezolizumab arm; Atezo + Beva = atezolizumab plus bevacizumab arm.
